# Identification and Fine Mapping of *Pi69*(t), a New Gene Conferring Broad-Spectrum Resistance Against *Magnaporthe oryzae* From *Oryza glaberrima* Steud

**DOI:** 10.3389/fpls.2020.01190

**Published:** 2020-08-07

**Authors:** Liying Dong, Shufang Liu, May Sandar Kyaing, Peng Xu, Didier Tharreau, Wei Deng, Xundong Li, Yunqing Bi, Li Zeng, Jing Li, Jiawu Zhou, Dayun Tao, Qinzhong Yang

**Affiliations:** ^1^ Agricultural Environment and Resources Research Institute, Yunnan Academy of Agricultural Sciences, Kunming, China; ^2^ Biotechnology Research Department, Ministry of Education, Mandalay, Myanmar; ^3^ Food Crops Research Institute/Yunnan Key Laboratory for Rice Genetic Improvement, Yunnan Academy of Agricultural Sciences, Kunming, China; ^4^ Key Laboratory of Tropical Plant Resources and Sustainable Use, Xishuangbanna Tropical Botanical Garden, Kunming, China; ^5^ Centre de Coopération Internationale en Recherche Agronomique pour le Développement (CIRAD), UMR BGPI, TA A 54 K, Montpellier, France; ^6^ BGPI, Univ Montpellier, CIRAD, INRA, Montpellier, SupAgro, Montpellier, France

**Keywords:** *Oryza glaberrima*, introgression line, *Magnaporthe oryzae*, *Pyricularia oryzae*, resistance gene, fine mapping

## Abstract

The discovery and deployment of new broad-spectrum resistance (*R*) genes from cultivated rice and its wild relatives is a strategy to broaden the genetic basis of modern rice cultivars to combat rice blast disease. *Oryza glaberrima* possessing many valuable traits for tolerance to biotic and abiotic stresses, is an elite gene pool for improvement of Asian cultivated rice. An introgression line IL106 derived from *O. glaberrima* (Acc. IRGC100137) confers complete resistance to *Magnaporthe oryza*e in blast nursery. Genetic analysis using 2185 BC_6_F_2_ progenies derived from a cross between IL106 and the recurrent parent Dianjingyou 1 showed that IL106 harbors a single dominant resistance gene against *M. oryzae* strain 09BSH-10-5A. This gene was preliminarily mapped on the long arm of chromosome 6 of rice in a region of ca. 0.9 cM delimited by two SSR markers (RM20650 and RM20701). In order to finely map this gene, 17,100 additional progenies were further analyzed. As a result, this gene was further narrowed down to a region flanked by two molecular markers STS69-15 and STS69-7, and co-segregated with 3 molecular markers, RM20676, STS69-21 and STS69-22 on the long arm of chromosome 6. Based on reference genome sequences, this *R* gene was mapped in silico in 76.1-Kb and 67.7-Kb physical intervals, and containing 4 and 3 NBS-LRR candidate genes in *O. sativa* cultivar Nipponbare and *O. glaberrima* cultivar CG14, respectively. Because no blast resistance gene was finely mapped in this physical interval before, this *R* gene was considered as not described yet and designated as *Pi69*(t), which is the first identified and finely mapped blast *R* gene from *O. glaberrima*, as far as we know. Evaluation of IL106 with 151 blast strains collected from 6 countries in Asia showed that 148 strains are avirulent on IL106, suggesting that *Pi69*(t) is a broad-spectrum blast *R* gene, and a promising resistant resource for improvement of Asian cultivated rice.

## Introduction

The African cultivated rice, *Oryza glaberrima* Steud., is well adapted for cultivation in West Africa (Linares, 2002; Sarla and Swamy, 2005), and possesses many valuable traits for tolerance to abiotic stresses, such as salinity, drought and strong weed competitiveness (Sarla and Swamy, 2005). *O. glaberrima* is also reported to have high level of resistance against several diseases and insect pests, such as *Rice yellow mottle virus* (Ndjiondjop et al., 1999; Pidon et al., 2017), bacterial leaf blight (Djedatin et al., 2011), blast (Silué and Notteghem, 1991; Rama Devi et al., 2015), green rice leafhopper (Fujita et al., 2010), as well as rice gall midge (Ukwungwu et al., 1998). Although it contains a narrow genetic base compared with other *Oryza* species (Wang et al., 2014; Meyer et al., 2016; Ndjiondjop et al., 2017), *O. glaberrima* is considered as an excellent gene reservoir for improvement of Asian cultivated rice, due to its useful traits of agronomic importance (Linares, 2002; Sarla and Swamy, 2005).

Rice blast, caused by the ascomycete fungus *Magnaporthe oryzae* (syn., *Pyricularia oryzae*) (Couch and Kohn, 2002), is one of the most destructive diseases for rice, and is responsible for significant yield losses under favorable environmental conditions worldwide (Ou, 1980; Savary et al., 2019). Rice-*M. oryzae* interactions follow the gene-for-gene relationship (Silué et al., 1992; Jia et al., 2000). Utilization of resistance (*R*) genes is one of the most economical, effective and environment-friendly approaches for blast control. However, the *R* genes of rice cultivars are often overcome shortly after their release, due to the emergence of strains of the pathogen virulent on certain *R* genes (Zeigler et al., 1994). Thus, it is necessary to mine new genes with broad spectrum of resistance against *M. oryzae* from diversities of rice species and use them in appropriate management strategies for durable control of blast in rice production (Zhu et al., 2000; Raboin et al., 2012; Sester et al., 2014). To date, over 100 blast *R* genes have been identified and mapped on different chromosomal regions of rice, through broad genetic and linkage analysis in the past decades (Ballini et al., 2008; Ashkani et al., 2016). These *R* genes have mainly been identified from *O. sativa*, and only 5 *R* genes originated from wild species of genus *Oryza*, including *Pi40* (*O. australiensis*), *Pi54rh* (*O. rhizomatis*), *Pi54* (*O. officinalis*), *Pi57*(t) (*O. longistaminata*), and *Pid3-A4* (*O. rufipogon*) (Jeung et al., 2007; Das et al., 2012; Lv et al., 2013; Devanna et al., 2014; Dong et al., 2017). *O. glaberrima* was domesticated from its wild progenitor *O. barthii* independently from *O. sativa* (Sweeney and McCouch, 2007). Although several studies previously reported that *O. glaberrima* expressed high level of resistance against rice blast (Silué and Notteghem, 1991; Rama Devi et al., 2015), no blast *R* gene locus was further identified and mapped yet. Whether blast *R* genes in *O. glaberrima* are different from *R* genes identified from other *Oryza* species so far remains unknown.

In order to discover useful genes of agronomic importance from *O. glaberrima*, a set of BC_5_F_4_ introgression lines (ILs) was constructed through successive backcross strategy between IRGC100137, an accession of *O. glaberrima* and *O. sativa* cultivar Dianjingyou 1 (DJY1), an *O. sativa* subsp. *japonica* cultivar, used as male recurrent parent (Xu et al., 2014). The ILs were evaluated for blast resistance in blast nursery in the field and by artificial inoculation with *M. oryzae* isolates in the greenhouse. Twelve ILs showing complete resistance to *M. oryzae* compared with the susceptible recurrent parent DJY1 were obtained. In this study, we describe the identification and fine mapping of a new blast resistance locus *Pi69*(t) from *O. glaberrima*.

## Materials and Methods

### Rice Materials and Mapping Population Construction

Resistant introgression line IL106 derived from *O. glaberrima* (accession No. IRGC100137) was crossed with a susceptible recurrent parent Dianjingyou 1 (DJY1) to generate BC_6_F_1_ seeds, the BC_6_F_1_ seeds were further sown and grown in a greenhouse to generate BC_6_F_2_ population for linkage and genetic analysis for resistance to rice blast. Resistant donor IL106, 10 monogenic lines (IRBLZ-Fu (*Piz*), IRBLZ5-CA (*Pi2*), IRBLzt-T (*Piz-t*), IRBL9-W (*Pi9*), IRBL5-M (*Pi5*), IRBLKH-K3 (*Pikh*), IRBL1-CL (*Pi1*), IRBL7-M (*Pi7*), IRBL20-IR24 (*Pi20*), and IRBLTA2-PI (*Pita2*)), as well as susceptible control cultivar Lijiangxintuanheigu (LTH) were used to test resistant/susceptible phenotypes to 151 *M. oryzae* strains.

### *M. oryzae* Cultivation and Spore Production

The *M. oryzae* isolate 09BSH-10-5A that is avirulent to IL106 and virulent to DJY1 was cultured on oatmeal medium (20 g of oatmeal, 15 g of agar, 10 g of sucrose, and 1 L of distilled water) for 7 days in the dark at 25°C. Then aerial mycelia were washed off by gentle rubbing with distilled water and paintbrush. The colony was then successively exposed to fluorescent light for 3 days to induce sporulation at 25°C. Conidia were harvested by softly scraping and flooding the medium surface with distilled water containing 0.01% Tween 20 detergent. The concentration of conidial suspension was adjusted to 50,000 conidia/ml for inoculation (Dong et al., 2017).

### Plant Planting and Pathotesting

The BC_6_F_2_ population seeds derived from the cross between IL106 and DJY1 were sown in plastic trays of 20×12×5 cm filled with paddy soil, and each tray was sowed with 95 germinated seeds. Seedlings were inoculated with *M. oryzae* strain 09BSH-10-5A by spraying at 4-leaf stage with 20 ml conidial suspension per tray. The inoculated rice plants were incubated overnight in a dark chamber at 25°C for 24 h with over 95% relative humidity, and then transferred back to the greenhouse. Lesion types on rice leaves were observed 6–7 days after inoculation and scored according to a standard reference scale (Silué et al., 1992). Plants scored from 1 to 3 were considered to be resistant and plants scored from 4 to 6 were considered to be susceptible. Furthermore, 151 *M. oryzae* isolates from 6 countries were used to test the resistant spectrum of *Pi69*(t) gene carrying in IL106 and 10 known blast *R* genes carrying in monogenic lines.

### Marker Development and Genetic Map Construction

Genomic DNA was extracted from fresh leaves of each plant following the method described by Edwards et al. (1991). A total of 229 SSR markers distributed evenly across all 12 rice chromosomes (McCouch et al., 2002) were used for identification of introgressed regions from *O. glaberrima*. Sequence-tagged site (STS) markers were developed within the critical region based on the sequence alignment of the genomic sequences of Nipponbare (*O. sativa*) and CG14 (*O. glaberrima*, http://plants.ensembl.org/Oryza_glaberrima/Info/Index).

PCR amplification conditions consisted of a denaturing step of 94°C/3 min, followed by 35 cycles of 94°C/30 s, annealing temperature 55°C/30 s, and 72°C/1 min, ending with an extension step of 72°C/7 min. Amplicons were separated by 8% polyacrylamide gel electrophoresis and detected by silver staining. Information of all primers used for gene mapping in this study is listed in **Table 1**. The genetic and linkage map of polymorphic markers was constructed using MAPMAKER/EXP 3.0 (Lander et al., 1987). The Kosambi mapping function was used to transform recombination frequency to genetic distance (cM).

**Table 1:** Summary of PCR primers used for linkage analysis.

Marker	Forward primer (5'-3')	Genomic position (bp) of Nipponbare^a^	Expected size (bp)^b^	Genomic position (bp) of CG14^c^	Expected size (bp)^d^	Annealing temperature(°C)
RM30	F: TGGGGTGGTTAGGCATCGTC	27253291-27253310	85	20426541-20426560	–	55
	R: CCTCACCACACGACACGAGC	27253375-27253356		not available		
RM345	F: ATGCAACCTCCTCTTCTCCA	30865862-30865881	136	23089611-23089630	143	55
	R: ATTGGTAGCTCAATGCAAGC	30865997-30865978		23089753-23089734		
RM20625	F: GGAGGGAGGAATGGGTACACG	28533451-28533471	182	21297908-21297928	107	55
	R: TTGAGAGTGAAACGAGAACCAACC	28533632-28533609		21298014-21297991		
RM20650	F: CGAGTGGATCAGCAAATCTACAGC	29161210-29161233	111	21803046-21803069	107	55
	R: CAGCATCAGGCTTGTGTTAATGG	29161320-29161298		21803152-21803130		
RM20676	F: GATCTCCACCACCTCCATCTCC	29885931-29885952	192	22383635-22383656	129	55
	R: CCTACATCAAGGCTCGCTACTGC	29886122-29886100		22383763-22383785		
RM20701	F: GAGAAGAAATTCAGAGAGCAGAGC	30349781-30349804	164	22767158-22767181	155	55
	R: CAACCACATGATCCATATGACG	30349944-30349923		22767312-22767290		
RM20661	F: GAACACATGACACCACCTTTGC	29479730-29479751	152	22031105-22031126	143	55
	R: GCGTTTCTCATTCTGTTCTTGC	29479881-29479860		22031247-22031226		
RM20674	F: CAACCCAACCCAACATCTGC	29782053-29782072	195	22299523-22299542	184	55
	R: CCTCTTGTCTTTGGAGGCCTTACC	29782247-29782224		22299706-22299683		
RM20678	F: CCGACCCATCAAACACAAATAGG	29976869-29976891	142	22466394-22466416	136	55
	R: CTTCTTCGGCTTCGCCTTCC	29977010-29976991		22466529-22466510		
STS69-21	F: GGTAGACAAGTTAACACCCAACCATGA	29899071-29899097	158	22395527-22395553	195	55
	R: GCACAGACAGGGGAGGAAGCAAAC	29899228-29899205		22395721-22395698		
STS69-7	F: ATCGGCCTGGTCTACTACGAGTAATC	29948504-29948529	136	22437870-22437895	131	55
	R: CCATTGATCAAATTCTACATGAATC	29948639-29948615		22438000-22437976		
STS69-15	F: CCTGTGTACGTGTGTTCTGTATGC	29872500-29872523	184	22370311-22370334	165	55
	R: CATCCACAAGCAGAGCTGGTC	29872683-29872663		22370475-22370455		
STS69-22	F: GCGCTGCGACGGAAAGAATA	29934780-29934799	147	22416918-22416899	150	55
	R: TCCGGCCTCTATATCCACAAAG	29934926-29934905		22417067-22417046		

F, forward; R, reverse.

^a^genomic position of each marker along chromosome 6 of O. sativa subsp. japonica cultivar Nipponbare (IRGSP1.0).

^b^expected size of PCR products in Nipponbare.

^c^genomic position of each marker along chromosome 6 of O. glaberrima cultivar CG14.

^d^expected size of PCR products in CG14.

### Physical Map Construction *In Silico* and Candidate Gene Annotation

To construct physical map of *Pi69*(t) *in silico* based on the reference genome sequence of *O. sativa* subsp. *japonica* cultivar Nipponbare, all molecular markers were anchored on chromosome 6 of Os-Nipponbare-Reference-IRGSP-1.0 pseudomolecules by BLAST (https://blast.ncbi.nlm.nih.gov/). To annotate the candidate *R* genes, both the 76.1-Kb and 67.7-Kb target regions in Nipponbare and CG14 respectively were analyzed by using the FGENSH platform (http://www.softberry.com/).

## Results

### Genetic Analysis for Blast Resistance in IL106

The resistant donor IL106, recurrent parent DJY1, BC_6_F_1_ plants from IL106/DJY1 and BC_6_F_2_ population were inoculated with 09BSH-10-5A (**Supplementary Figures S1A, B**). The resistant donor IL106 and BC_6_F_1_ plants showed complete resistance, and recurrent parent DJY1 was susceptible to 09BSH-10-5A. The segregation of resistant and susceptible progenies among 2185 BC_6_F_2_ individuals fitted with an expected 3:1 ratio (resistant/susceptible: 1664/521, *χ* ^2^ = 1.556, *P* = 0.212), indicating that a single dominant *R* gene from IL106 confers complete resistance to *M. oryzae* strain 09BSH-10-5A.

### Identification and Mapping of *R* Gene Locus in IL106

To identify and map the *R* locus in IL106, a total of 217 SSR markers distributed evenly across all 12 rice chromosomes were used to determine the polymorphism between resistance donor IL106 and recurrent parent DJY1. As expected, a large majority of markers were monomorphic between IL106 and its recurrent parent. Three SSR markers, RM345 on chromosome 6, RM6329 on chromosome 3, and RM3702 on chromosome 8, showed polymorphism between IL106 and DJY1, suggesting that three introgression fragments from *O. glaberrima* possessed in IL106. In order to verify the linkage relationship between these 3 SSR loci and *R* gene in IL106, 94 random susceptible individuals from BC_6_F_2_ population inoculated with 09BSH-10-5A were genotyped with these 3 SSR markers. The results showed that the severe segregating distortion (92 homozygotes of susceptible allele to 2 heterozygotes) was only detected for RM345, implying linkage between the *R* gene and this marker which located on chromosome 6.

**Figure 1:**
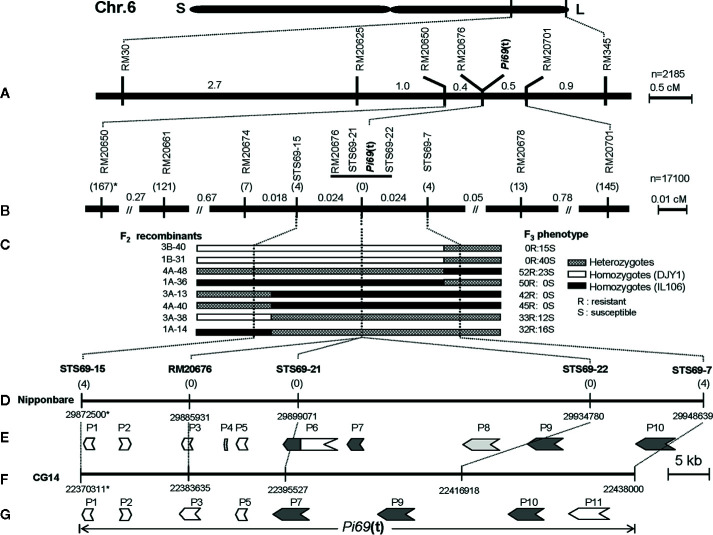
Genetic and physical maps of *Pi69*(t) locus on rice chromosome 6. **(A)** A genetic map of *Pi69*(t) locus. Map distances are in cM. **(B)** An integrated fine genetic map of *Pi69*(t) locus on chromosome 6, *: the numbers in parentheses under the markers present the number of recombinants between marker loci and *Pi69*(t); **(C)** The genotyping and phenotyping of key recombinants located between two markers STS69-15 and STS69-7. **(D)** Physical map of the *Pi69*(t) locus based on the reference genome sequence of *O. savita* cultivar Nipponbare. *: chromosomal position of markers on genomic sequence of chromosome 6 of Nipponbare; **(E)** Predicted candidate *R* genes for *Pi69*(t) in Nipponbare (*O. sativa*). **(F)** Physical map of the *Pi69*(t) locus based on the reference genome sequence of *O. glaberrima* accession CG14. *: chromosomal position of markers on genomic sequence of chromosome 6 of CG14; **(G)** Predicted candidate *R* genes for *Pi69*(t) in CG14 (*O. glaberrima*).

To determine the *O. glaberrima* introgression length of the *R* gene region in IL106, 27 SSR markers located on the long arm of chromosome 6 were selected to survey the polymorphism between IL106 and DJY1. The result showed that the introgression fragment was located between SSR markers RM30 and RM345, and 4 SSR markers (RM20625, RM20650, RM20676, and RM20701) within this interval were also polymorphic. Subsequently, the mapping population consisting of 2,185 BC_6_F_2_ plants was genotyped with the two SSR markers RM30 and RM345, and the recombinants were further genotyped with 4 SSR markers (RM20625, RM20650, RM20676, and RM20701) to map the *R* gene location. Taken together, the *R* locus was mapped to a 0.9 cM region flanked by RM20650 and RM20701 on the long arm of chromosome 6, and co-segregated with RM20676 (**Figure 1A**).

### Fine Mapping of *R* Gene Locus in IL106

To further map this *R* gene locus, 17,100 additional BC_6_F_2_ plants were genotyped with two flanking markers RM20650 and RM20701. All the 312 recombinants were then phenotyped for resistance to *M. oryzae* strain 09BSH-10-5A. As a result, 167 and 145 recombinants were detected between phenotypes (R or S) and RM20650 and RM20701 genotypes, respectively (**Figure 1B**). Meanwhile, new SSR markers located in RM20650-RM20701 interval were surveyed for polymorphism between IL106 and DJY1, and 3 polymorphic SSR markers RM20661, RM20674, and RM20678 were obtained. Then, the 312 recombinants were genotyped with these new SSR markers and RM20676. The recombination events btweeen the *R* locus and RM20661, RM20674, RM20676, and RM20678 were 121, 7, 0, and 13, respectively (**Figure 1B**), The *R* locus was linked to RM20661 and RM20674 by a genetic distance of ca. 0.71, and 0.041 cM, co-segregated with RM20676, and linked to RM20678 by 0.076 cM on the other side, respectively.

To further finely map the *R* locus, 24 STS markers were developed based on the genome sequences of Nipponbare and CG14. Five STS markers polymorphic between IL106 and DJY1 were used to genotype the 7 and 13 recombinants at RM20674 and RM20678 loci, respectively. As a result, 4 recombinants were detected at STS69-15 locus on RM20674 side, and 4 recombinants were detected at STS69-7 locus on RM20678 side. No recombinants were identified at RM20676, STS69-21, and STS69-22 loci. These results indicated that the *R* gene locus was narrowed down to the region flanked by STS69-15 and STS69-7, and co-segregated with 3 molecular markers RM20676, STS69-21, and STS69-22 (**Figure 1B**). The genotypes and phenotypes of 8 recombinants between STS69-15 and STS69-7 are shown in **Figure 1C**.

### Differentiation Between *R* Gene Carrying in IL106 and *Pi-tq1* in Teqing

The blast resistant gene *Pi-tq1* from *indica* cultivar Teqing was previously mapped to a 4.24 Mb physical interval flanked by two RFLP markers, RZ682 and RZ508 (Tabien et al., 2000) that spanned over the *R* locus described here on the long arm of chromosome 6. To distinguish these two genes, IL106 and Teqing were inoculated with 3 *M. oryzae* strains (09BSH-10-5A, BS139, and HN09-1C-7) that are avirulent on IL106, but virulent on DJY1. The result showed that Teqing was resistant to both 09BSH-10-5A and BS139, but susceptible to HN09-1C-7 (**Supplementary Figure S1C**), suggesting that the *R* gene in IL106 could be different from *Pi-tq1*, because of their distinct reactions to HN09-1C-7. To demonstrate that the resistance of HN09-1C-7 is controlled by the same *R* gene in IL106, this strain was inoculated to 191 F_2_ progenies from the cross between IL106 and DJY1. The numbers of resistant and susceptible individuals were 149 and 42, respectively and fitted to a 3:1 ratio (*P*=0.337), confirming that IL106 possesses a single resistance gene to the strain HN09-1C-7. Genotyping of 42 susceptible and 4 resistant individuals with 4 molecular markers (RM30, RM345, STS69-7, and STS69-15) linked with *Pi69*(t), identified 4 and 3 recombinants at RM30 and RM345 loci, respectively, and no recombinants at both STS69-7 and STS69-15 loci (**Supplementary Figure S2**), indicating that the gene conferring resistance to HN09-1C-7 in IL106 was mapped to the same chromosomal region as that *R* gene conferring resistance to 09BSH-10-5A, and the resistance to both 09BSH-10-5A and HN09-1C-7 is controlled by the same *R* gene in IL106. Taken together, all these data confirmed that *R* locus in IL106 is different from *Pi-tq1*, due to their distinct reactions to *M. oryzae* strain HN09-1C-7. Because no blast *R* gene was finely mapped in chromosomal region flanked by STS69-15 and STS69-7 on chromosome 6 of rice to date, this major *R* gene carrying in IL106 from *O. glaberrima* was considered as a new gene and was tentatively designated as *Pi69*(t).

### Resistance Spectrum of *Pi69*(t)

To determine the resistance spectrum of *Pi69*(t), IL106, and other 10 monogenic lines carrying broad-spectrum *R* genes were inoculated and assessed with 151 *M. oryzae* strains from Cambodia (16 strains), Laos (20 strains), Myanmar (4 strains), Thailand (20 strains), Vietnam (18 strains), and China (77 strains). IL106 was resistant to all strains from Cambodia and Laos, and susceptible to only 3 strains from China, Thailand and Vietnam (YX162, TH451, and VN4118; **Supplementary Table S1**). IL106 also showed broader resistant spectrum compared with nine monogenic lines carrying different known *R* genes, except for the line IRBL9-W carrying *Pi9* that was resistant to all tested strains. These results suggest that *Pi69*(t) gene could confer broad-spectrum resistance against *M. oryzae* in IL106.

### *In Silico* Physical Map Construction of *Pi69*(t) Gene Locus

To construct the physical map of *Pi69*(t) locus *in silico*, all the molecular markers closely linked to *Pi69*(t) were anchored to the genome sequences of both *O. sativa* subsp. *japonica* cultivar Nipponbare (IRGSP1.0) and *O. glaberrima* cultivar CG14 (AGI1.1) through BLAST analysis (http://plants.ensembl.org/). The two flanking markers and 3 co-segregating markers were anchored to the target region (**Figures 1D, F**). The physical distance between two closest flanking markers STS69-15 and STS69-24 was about 76.1 Kb (genomic position: 29872500-29948639) in Nipponbare, and 67.7 Kb (genomic position: 22370311- 22438000) in CG14.

Both the target genome sequences from Nipponbare and CG14 were annotated through the bioinformatics platform FGENSH (http://www.softberry.com). The annotation showed that 8 and 10 genes (named tentatively from *P1* to *P11*) were predicted in Nipponbare and CG14, respectively (**Figures 1E, G**). Among all these annotated genes, *P4* and *P8* genes were absent in CG14, while *P11* gene was absent in Nipponbare. Among these predicted genes, both *P1*(*LOC_Os6g49300*) and *P3*(*LOC_Os6g49320*) encode the putative genes homologous with glycosyltransferase; *P2*(*LOC_Os6g49310*) encodes a gene homologous to MATE efflux family protein; *P4* (*LOC_Os6g49330*) annotated in Nipponbare only is an uncharacterized protein; *P5* (*LOC_Os6g49340*) encodes a F-box and DUF domain containing protein; P6 (*LOC_Os6g49350*) and *P11* encode a retrotransposon in Nipponabre and CG14 respectively; the remaining four genes (*P7* (*LOC_Os6g49360*), *P8* (*LOC_Os6g49380*), *P9* (*LOC_Os6g49390*), and *P10* (*LOC_Os6g49420*)) were predicted to be typical *R* genes encoding protein with the conserved structure of nucleotide-binding site and leucine-rich repeat (NBS-LRR; **Figures 1E, G**). In comparison with *P7* in CG14, there are two genes (*P6* and *P7*) in Nipponbare caused by an insertion of retrotransposon. Amino acids analysis among P7 in CG14, P6, and P7 in Nipponbare showed that the amino acid sequence on the 3’ side of P6 in Nipponbare has high similarity with those in P7 of CG14, and *P7* in Nipponbare encoded only a truncated NBS-LRR protein compared with its P7 allele in CG14.

### Evidence of *O. glaberrima* Genome Fragment Integration

**Figure 2:**
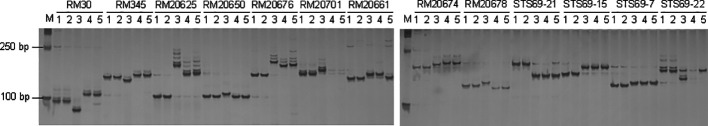
Confirmation of the origin of the introgression of the *Pi69*(t). Thirteen molecular markers linked with *Pi69*(t) were used to amplify the DNA fragments of the *O. glaberrima* (IRGC100137) donor parent (1), the introgression line IL106 (2), the recurrent parent DJY1 (3), and two *indica* cultivars: R498 (4), and Teqing (5). M, molecular weight marker DL2000. The PCR products were separated by 8% polyacrylamide gel.

To validate whether the fragment carrying *Pi69*(t) in IL106 was integrated from *O. glaberrima*, the introgression line IL106, the original *O. glaberrima* accession IRGC100137, the recurrent parent *japonica* cultivar DJY1, as well as two *indica* cultivars R498 and Teqing, were genotyped with 13 molecular markers used for mapping *Pi69*(t). The results showed that the size of all the DNA fragments amplified from IL106 were the same as those from IRGC100137 (**Figure 2**). Meanwhile, these molecular markers were polymorphic among line IL106, and all rice cultivars (DJY1, R498 and Teqing).

## Discussion

The discovery and deployment of broad-spectrum *R* genes from a large number of cultivated rice varieties and its wild relatives is an effective strategy to broaden the genetic basis of resistance of modern rice cultivars, to cope with the diversity and variability over time of pathogen population in rice production (Jeung et al., 2007; Su et al., 2015; Deng et al., 2017). Several incompatibility barriers such as pre- and post-fertilization barriers, hybrid sterility between Asian cultivated rice and its wild and cultivated relatives, however, have been hindering the utilization of favorable genes controlling important agronomic traits (Brar and Khush, 1997; Xu et al., 2014; Brar and Khush, 2018). Construction of introgression lines of these relatives with Asian cultivated rice cultivars has been proved to be one of the effective measures for further discovery and use of favorable genes from the wild species for rice breeding, and several genes conferring resistance to biotic stresses from wild species were identified in introgression lines (Brar and Khush, 1997; Gutierrez et al., 2010; Rama Devi et al., 2015; Brar and Khush, 2018). Extensive studies on exploiting and identification of blast resistance genes have been conducted, mainly focusing on the *O. sativa* and wild relatives. Relatively few investigations have been performed to identify new blast *R* genes in *O. glaberrima* (Silué and Notteghem, 1991; Rama Devi et al., 2015). In this study, we have successfully identified and finely mapped *Pi69*(t), the first blast *R* gene from *O. glaberrima* by using an introgression line IL106 derived from *O. glaberrima*. *Pi69*(t) confers a broad-spectrum resistance to *M. oryzae* diverse strains from 6 Asian countries, indicating that *Pi69*(t) is a promising resistance resource for improvement of Asian cultivated rice for resistance to rice blast.

Over 20 blast major *R* genes have been identified and mapped on rice chromosome 6, and the majority of them were mapped proximal to the centromere. The cloned *R* genes *Piz-t*, *Pi2*, *Pi9*, *Pi50*, and *Pigm* are members of the multigene family *Pi2*/*Pi9* locus located on the short arm, while the *Pid-2*, *Pid3/Pi25*(t), and *Pid3-A4* are located on the long arm (Chen et al., 2006; Shang et al., 2009; Chen et al., 2011; Lv et al., 2013). Using recombination inbred lines derived from a cross between Lemont and Teqing, Tabien et al. (2000) identified and mapped 3 blast resistance genes from Teqing (*Pi-tq1*, *Pi-tq2*, and *Pi-tq3*). Among them, *Pi-tq1* was also mapped to chromosome 6 of rice but located proximal to telomeric side. Two flanking markers RZ682 and RZ508 defined a larger physical region of *Pi-tq1* locus of around 4.24 Mb covering *Pi69*(t) locus. Although *Pi69*(t) could be differentiated from *Pi-tq1* by using *M. oryzae* strain HN09-1C-7, due to their distinct reactions to this strain, whether *Pi69*(t) is allelic, or closely linked to *Pi-tq1* remains to be determined through allelism test or fine mapping of *Pi-tq1*.

Pyramiding of *R* genes with different resistance specificity in the same cultivar is one effective measure to broaden the resistance spectrum against *M. oryzae*, and development of polymorphic molecular markers is the prerequisite to stack target genes into one cultivar with marker-assisted selection method (Hittalmani et al., 2000). The molecular markers developed in this study, tightly linked to *Pi69*(t), showed good polymorphisms among 5 tested rice lines/cultivars belonging to *indica* or *japonica* types. These markers are good candidates for pyramiding of *Pi69*(t) with other *R* genes for improvement of Asian cultivated cultivars in disease-resistant rice breeding program.

Most of cloned resistance genes from plants encode NBS-LRR like proteins that directly or indirectly recognize the pathogen effectors to trigger host defense responses (Dangl and Jones, 2001). Almost all the cloned rice *R* genes to blast encode NBS-LRR like proteins, except for *Pid2*, *pi21*, and *Ptr* genes, which encode a B-lectin receptor kinase, a proline-rich protein, and an atypical protein with amardillo repeat domain, respectively (Lv et al., 2013; Su et al., 2015; Ashkani et al., 2016; Deng et al., 2017; Zhao et al., 2018). *O. glaberrima*-derived *Pi69*(t) gene was located in a region containing a cluster of NBS-LRR like genes. These genes are potential or promising candidates for *Pi69*(t).

## Data Availability Statement

All datasets presented in this study are included in the article/**Supplementary Material**

## Author Contributions

QY, DaT, LD, SL, PX, and DiT: Conceived idea and designed research. QY, DaT, and DiT wrote the manuscript. LD, SL, MK, QY, WD, XL, YB, LZ, JL, and JZ: Performed experiments and analyzed data. All authors contributed to the article and approved the submitted version.

## Funding

This work was supported by the National Natural Science Foundation of China (31860524) to LD, the Applied Basic Research Programs of Yunnan Academy of Agricultural Sciences (YJZ201803) to LD, the National Natural Science Foundation of China (31560493) to QY, the Applied Basic Research Programs of Yunnan Academy of Agricultural Sciences (YJM201707) to SL, the Key Research and Development Program of Yunnan Province (2019IB007), the Scientific Observing and Experimental Station of Crops Pests in Kunming, the Ministry of Agricultural, and the Rural Affairs of China.

## Conflict of Interest

The authors declare that the research was conducted in the absence of any commercial or financial relationships that could be construed as a potential conflict of interest.
