# Supplementary material for: Identification and Fine Mapping of Pi69(t), a New Gene Conferring Broad-Spectrum Resistance Against Magnaporthe oryzae From Oryza glaberrima Steud
Source: Front Plant Sci. 2020 Aug 7;11:1190. doi: 10.3389/fpls.2020.01190 (PMC7426465; doi:10.3389/fpls.2020.01190)
Supplement: Supplementary file 1 [file DataSheet_1.pdf]

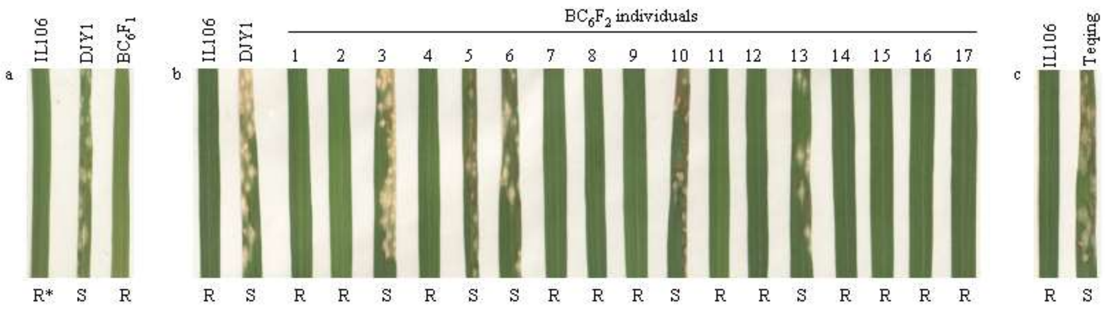

Supplementary Figure S1 Phenotypes of parents IL106 and Dianjingyou 1, BC_6F_1, BC_6F_2 and *indica* cultivar Teqing to *M. oryzae*. a Phenotypes of two parents IL106, Dianjingyou 1 and their BC_6F_1 to *M. oryzae* strain 09BSH-10-5A; b Phenotypes of IL106, Dianjingyou 1 and 17 BC_6F_2 progenies to *M. oryzae* strain 09BSH-10-5A; c Phenotypes of IL106 and Teqing to *M. oryzae* strain HN09-1C-7. *: R=resistant, S=susceptible.
