# Supplementary material for: Identification and Fine Mapping of Pi69(t), a New Gene Conferring Broad-Spectrum Resistance Against Magnaporthe oryzae From Oryza glaberrima Steud
Source: Front Plant Sci. 2020 Aug 7;11:1190. doi: 10.3389/fpls.2020.01190 (PMC7426465; doi:10.3389/fpls.2020.01190)
Supplement: Supplementary file 2 [file DataSheet_2.pdf]

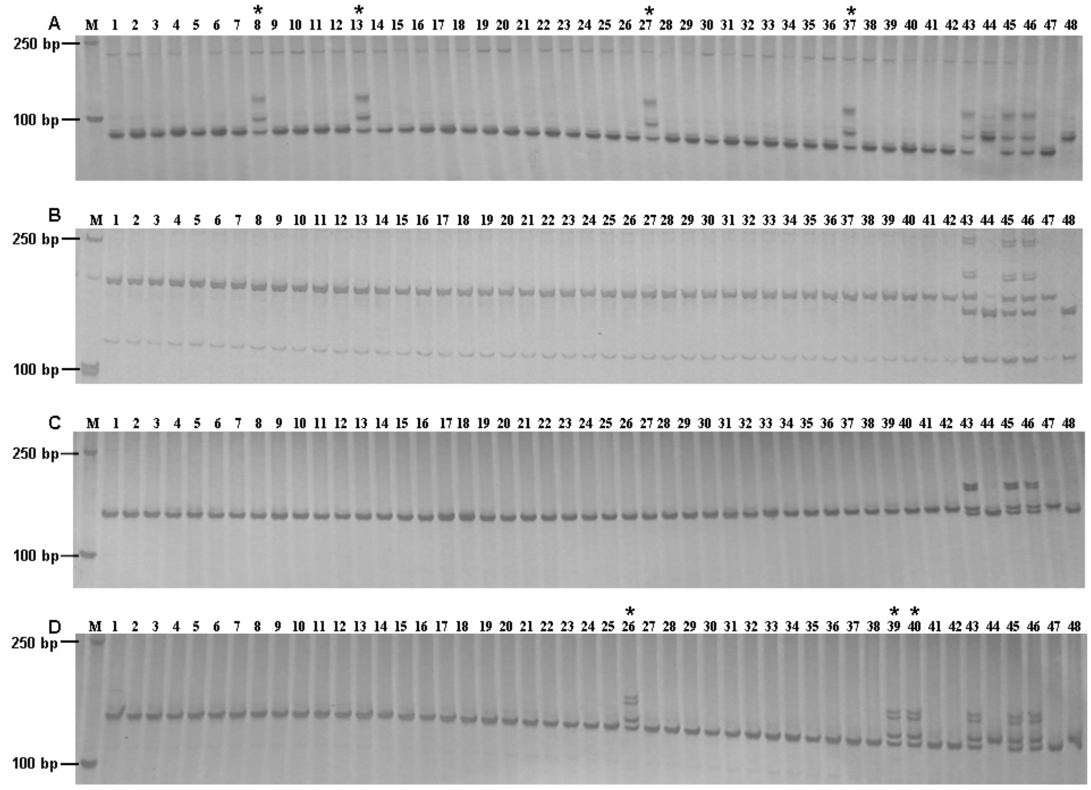

Supplementary Figure S2 Genotyping of 46 F_2 individuals from the cross between IL106 and DJY1 with 4 molecular markers, RM30 (A), STS69-15 (B), STS69-7 (C) and RM345 (D). *: recombinants; M: molecular weight marker, DL2000; lanes 1 to 42: susceptible individuals; lanes 43 to 46: resistant individuals, lane 47: susceptible parent DJY1, lane 48: resistant donor, IL106
